# Cost-effectiveness of catheter-based radiofrequency renal denervation for the treatment of uncontrolled hypertension: an analysis for the UK based on recent clinical evidence

**DOI:** 10.1093/ehjqcco/qcae001

**Published:** 2024-01-09

**Authors:** Andrew S P Sharp, Khoa N Cao, Murray D Esler, David E Kandzari, Melvin D Lobo, Roland E Schmieder, Jan B Pietzsch

**Affiliations:** Department of Cardiology, University Hospital of Wales and Cardiff University, Cardiff, CF14 4XW, UK; Wing Tech Inc., Menlo Park, CA 94025, USA; Human Neurotransmitters Laboratory, Baker IDI Heart and Diabetes Institute, Melbourne, VIC 3004, Australia; Department of Interventional Cardiology, Piedmont Heart Institute, Atlanta, GA 30309, USA; Bart’s Blood Pressure Clinic, Bart’s Health NHS Trust, London E1 2ES, UK; Department of Nephrology and Hypertension, University Hospital Erlangen, 91054 Erlangen, Germany; Wing Tech Inc., Menlo Park, CA 94025, USA

**Keywords:** Hypertension, Denervation, Radio frequency ablation, Cost–benefit analysis, England

## Abstract

**Aims:**

Catheter-based radiofrequency renal denervation (RF RDN) has recently been approved for clinical use in the European Society of Hypertension guidelines and by the US Food and Drug Administration. This study evaluated the lifetime cost-effectiveness of RF RDN using contemporary evidence.

**Methods and results:**

A decision–analytic model based on multivariate risk equations projected clinical events, quality-adjusted life years (QALYs), and costs. The model consisted of seven health states: hypertension alone, myocardial infarction (MI), other symptomatic coronary artery disease, stroke, heart failure (HF), end-stage renal disease, and death. Risk reduction associated with changes in office systolic blood pressure (oSBP) was estimated based on a published meta-regression of hypertension trials. The base case effect size of −4.9 mmHg oSBP (observed vs. sham control) was taken from the SPYRAL HTN-ON MED trial of 337 patients. Costs were based on National Health Service England data. The incremental cost-effectiveness ratio (ICER) was evaluated against the UK National Institute for Health and Care Excellence (NICE) cost-effectiveness threshold of £20 000–30 000 per QALY gained. Extensive scenario and sensitivity analyses were conducted, including the ON-MED subgroup on three medications and pooled effect sizes. RF RDN resulted in a relative risk reduction in clinical events over 10 years (0.80 for stroke, 0.88 for MI, 0.72 for HF), with an increase in health benefit over a patient's lifetime, adding 0.35 QALYs at a cost of £4763, giving an ICER of £13 482 per QALY gained. Findings were robust across tested scenarios.

**Conclusion:**

Catheter-based radiofrequency RDN can be a cost-effective strategy for uncontrolled hypertension in the UK, with an ICER substantially below the NICE cost-effectiveness threshold.

## Graphical Abstract

**Graphical Abstract:**
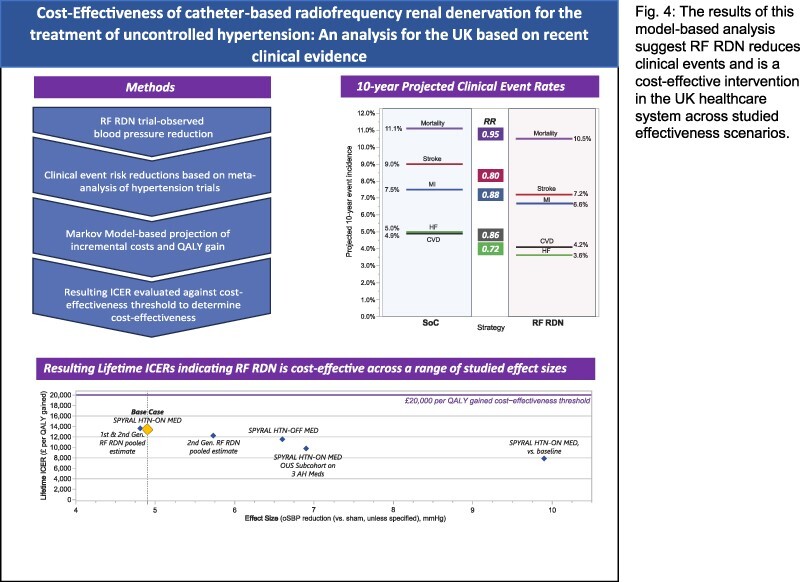
The results of this model-based analysis suggest RF RDN reduces clinical events and is a cost-effective intervention in the UK healthcare system across studied effectiveness scenarios.AH: anti-hypertensive; CVD: cardiovascular death; ESRD: end-stage renal disease; HF: heart failure; ICER: incremental cost-effectiveness ratio; MI: myocardial infarction; Mortality = all-cause death; oSBP: office-based systolic blood pressure; OUS: treated outside the United States; RF RDN: radiofrequency renal denervation; RR: relative risk; SoC: standard of care; QALY: quality-adjusted life year.

Key Learning Points What is already known:

- Hypertension remains the major cause of avoidable death worldwide.
- Catheter-based renal denervation is now approved for clinical use in the European Society of Hypertension guidelines of 2023 and by the US Food and Drug Administration.
- Sham-controlled trials and long-term registries show reductions in office systolic blood pressure, an accepted predictor of long-term clinical outcomes.

What this study adds:

- Radiofrequency renal denervation meets the standard for cost-effectiveness over a lifetime horizon, as defined by the UK National Institute for Health and Care Excellence.
- This applies across a range of scenarios incorporating a conservative interpretation of the clinical evidence available.

## Introduction

Hypertension remains a significant clinical challenge as a leading cause of morbidity and mortality worldwide.^1,2^ Radiofrequency renal denervation (RF RDN) is a minimally invasive, catheter-based therapy, which ablates the renal nerves to interrupt sympathetic signals to and from the kidneys and has been shown to lower systolic blood pressure (SBP) to a significantly greater extent than sham therapy without major adverse events.^3^ Since its introduction, RF RDN has been refined to deliver radiofrequency energy simultaneously to all four renal artery quadrants, reducing procedural time and potentially improving efficacy.^4^

The latest generation of pivotal studies, including the SPYRAL HTN-ON MED and HTN-OFF MED studies, demonstrated the safety and effectiveness of RF RDN in sham-controlled randomized controlled trials.^5–7^ In the SPYRAL HTN-ON MED trial (full cohort, combining results of the pilot and extension studies), which examined therapy in the presence of antihypertensives, 337 participants were randomized to either RF RDN treatment (*n* = 206) or sham control (*n* = 131). Subjects who received RF RDN reported a 9.9 mmHg reduction in office-based SBP (oSBP), while those in the sham cohort reported a 5.0 mmHg reduction, yielding a statistically significant reduction of 4.9 mmHg in the treated group compared with sham.^6^ In the SPYRAL HTN-OFF MED study, which examined therapy in the absence of antihypertensives, the RF RDN cohort reported a reduction of 9.6 mmHg in oSBP compared with 3.5 mmHg in the sham cohort, yielding a reduction of 6.5 mmHg in the treated group over and above sham.^5^ The clinical trial findings for RF RDN over the past decade have also been supported by evidence from the Global Symplicity Registry (GSR), a prospective, multicentre, open-label registry that has collected data on >3000 subjects, reporting sustained, long-term efficacy without major safety concerns through 3 years and beyond^8^ with early data supporting longevity of effect out to 9 year follow-up.^9^ There is an increasing recognition that RF RDN has promise as an adjunctive treatment option to lifestyle modification and anti-hypertensive drug therapy in uncontrolled hypertension^10,11^ and as such is now recommended for clinical use in the new update of the 2023 European Society of Hypertension Management of Arterial Hypertension Guidelines.^12^ Based on the same body of evidence, the US Food and Drug Administration approved RF RDN in November 2023 for clinical use in the USA, substantially increasing global access to this therapeutic option. Both of these developments contribute to a growing interest in understanding the health–economic implications of RF RDN therapy adoption.

The cost-effectiveness of renal denervation for resistant hypertension has previously been reported based on the results of the open label Symplicity HTN-2 trial using the first-generation Symplicity Flex catheter.^13,14^ The current study sought to expand upon these analyses to determine the cost-effectiveness of RF RDN for the treatment of the broader uncontrolled hypertension patient group, reflecting contemporary data and evidence from the new generation of clinical studies that utilized different trial designs and procedural technologies/techniques. The UK healthcare system was chosen as the setting for the analysis as it represents a European healthcare system where cost-effectiveness considerations are integral to the adoption of new technologies, and this analysis deploys methodologies used by the UK National Institute for Health and Care Excellence (NICE), with an intent to use the developed analysis framework also in future studies in other European countries and beyond.

## Methods

A decision–analytic, state-transition Markov model was used to examine the health benefits and costs of RF RDN. The analysis model was built on a previously published model that was updated and expanded for the current study.^14^ While the same analytical structure was maintained, the new model included an ability to adjust multivariate risk functions to explore the effect of variation in baseline event risk, a consideration of relative risk (RR) reductions from specific reductions in oSBP based on published meta-regression data, and the use of contemporary treatment and event cost data. The use of oSBP as the effect measure was pre-specified as underlying risk models and meta-analyses rely on this measure.

In the base case analysis, the treatment cohort received RF RDN with the Symplicity SPYRAL™ renal denervation system (Medtronic Inc., Santa Rosa, CA, USA), while the sham control cohort received a renal angiogram alone. Blinding was maintained and effective.^6^ Per trial protocol, subjects were standardized on a medication regimen of one, two, or three antihypertensive medication classes prior to randomization. The model projected outcomes for stroke, coronary heart disease (CHD), myocardial infarction (MI), heart failure (HF), end-stage renal disease (ESRD), cardiovascular death (CVD), and all-cause death (ACD). Transition probabilities were derived from multivariate risk equations from large cohort studies, including the Framingham Heart Study. Model inputs such as costs and utilities were determined from published literature and micro-costing exercises.

### Model structure and framework

**Figure 1:**
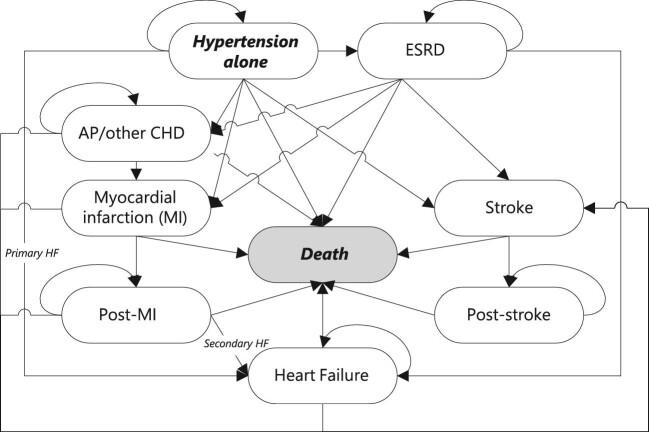
Model schematic, showing transitions among primaryg all modelled health states, based on Geisler *et al*. 2012.^14^ AP, angina pectoris; CHD, coronary heart disease; MI, myocardial infarction; ESRD, end-stage renal disease.

The Markov model was constructed from a UK healthcare payer perspective [National Health Service (NHS) England] with a lifetime horizon, a cycle length of 1 month with half-cycle correction and a discounting of costs and effects at 3.5% per annum as per NICE guidelines.^15^ Clinical disease progression was modelled using 33 health states to reflect both primary (e.g. MI, stroke, HF, angina pectoris, and ESRD) and secondary health states (e.g. stroke post-MI or HF post-ESRD) (*Figure 1*). An in-depth description of the health states is given in Supplementary material online, *S1*. The model was constructed in Microsoft Excel (Microsoft, Redmond, WA, USA). Statistical analyses were performed in JMP Pro 16 (SAS Institute, Cary, NC, USA). The analysis followed the Consolidated Health Economic Evaluation Reporting Standards, as documented in Supplementary material online, *S13*.^16^

### Transition probabilities and relative risk reductions

Transition probabilities to different health states, reflecting the development of hypertension sequelae, were based on multivariate risk equations from large cohort studies and took into account the SPYRAL HTN-ON MED trial cohort characteristics (Supplementary material online, *S2*).^17–23^ The risk of stroke, CHD and HF were based on the Framingham risk equations.^17–19^ In the absence of an MI-specific risk function from the Framingham equations, the risk of MI was based on the Prospective Cardiovascular Münster Heart Study risk equation.^20^ The risk of ESRD was calculated from National Health and Nutrition Examination Surveys risk equations.^24^ Condition-specific mortality rates were obtained from large-scale British studies (Supplementary material online, *S3*).^14,25–31^ Multivariate risk equations could be adjusted with a hazard ratio to vary baseline event risk. Meta-regression data from 47 randomized controlled trials specifically designed to assess the effects of blood pressure reduction in hypertension patients^32^ were used to calculate RR reductions for clinical events (Supplementary material online, *S4*).

### Clinical data

The SPYRAL HTN-ON MED trial was selected for the base case analysis as it is the most contemporary large-scale randomized sham-controlled trial for the current generation of RF RDN devices studied in a target population of interest—uncontrolled hypertension receiving one to three antihypertensive medications. The reduction in oSBP and patient demographics in the base case were obtained from the full cohort of that trial. Additional scenario analyses were carried out accounting for the oSBP reduction observed for the SPYRAL HTN-ON MED trial subgroup of patients on three antihypertensive medications treated outside the USA (a sub-cohort that could be considered analogous to a resistant hypertension cohort), for the oSBP reduction observed in the HTN-OFF MED trial, and for the meta-analytic effect size calculated from all sham-controlled first- and second-generation sham-controlled RF RDN device trials.

Details about these studies can be found in their respective published clinical papers.^5–7,33^ In the SPYRAL HTN-ON MED trial study, participants were, on average, 55 years of age with a baseline oSBP of 163 mmHg and, according to the protocol, with a prescription of one to three (mean 1.9) medications.^6,7^ At 6-month follow-up for SPYRAL HTN-ON MED trial, RF RDN reduced oSBP by 9.9 mmHg in the therapy group and 5.0 mmHg in the sham group, leading to a statistically significant 4.9 mmHg reduction compared with sham.^6,7^ The SPYRAL HTN-ON MED trial study sub-cohort of patients on three antihypertensive medications treated outside the USA considered an observed reduction against sham of 6.9 mmHg.^33^ In the HTN-OFF MED study, participants were, on average, 52.5 years with a baseline oSBP of 162.8 mmHg, and did not receive any anti-HTN medication.^5^ RF RDN reduced oSBP by 9.2 mmHg in the therapy group and 2.5 mmHg in the sham group, yielding a reduction of 6.5 mmHg compared with sham.^5^ The pooled effect sizes derived from the comprehensive body of first- and second-generation RF RDN studies were 4.8 and 5.7 mmHg for first- and second-generation and second-generation devices, respectively (Supplementary material online, *S5*).^34–36^ Effect sizes were assumed to be maintained over lifetime, as supported by an emerging body of evidence reporting maintained treatment effect out to nearly 10 years.^9,37–39^

### Costs and health-related quality of life

Input costs for medications and management of clinical events were obtained from published literature and relevant UK guidelines. The cost of RF RDN treatment was assessed using a micro-costing approach that considered pre-procedure and procedure costs, including personnel, device and catheterization laboratory overhead cost, as well as one night of hospital stay. All costs were expressed in the British Pound (£) and adjusted to 2022 values using Consumer Price Inflation (Health) values from the UK Office of National Statistics.^40^ Health state-specific utilities were based on published literature and were age-adjusted using data for the UK.^41^ Mortality rates were obtained from the latest English lifetables,^42^ and post-event survival based on data from published literature.^6,7,15,43–50^ See *Table 1* for key input parameters.

**Table 1:** Key input parameters

Parameter	Value	Distribution	SE	Source
Age	55.0 years	Normal	0.53	SPYRAL HTN-ON MED trial full cohort, Kandzari *et al*. 2023^6^; Kandzari *et al*. 2022^7^
Gender (% female)	19.9%	Beta	0.022	SPYRAL HTN-ON MED trial full cohort, Kandzari *et al*. 2023^6^; Kandzari *et al*. 2022^7^
Baseline systolic BP	163 mmHg	Normal	0.40	SPYRAL HTN-ON MED trial full cohort, Kandzari *et al*. 2023^6^; Kandzari *et al*. 2022^7^
Treatment effect	4.9 mmHg	Normal	0.54	SPYRAL HTN-ON MED trial full cohort, Kandzari *et al*. 2023^6^; Kandzari *et al*. 2022^7^
Discount rate (costs)	3.50% p.a.			NICE 2012^15^
Discount rate (effects)	3.50% p.a.			NICE 2012^15^
Costs (one time/annual)				
Hypertension (year one+)	£279	Gamma	£28	NICE Guidelines 2019^43^
Stroke (acute)	£15 327	Gamma	£1533	NICE Guidelines 2019,^43^ NHS Sentinel Stroke National Audit 2016^44^
Stroke (year one)	£9 926	Gamma	£1083	
Stroke (year two+)	£5 672	Gamma	£567	
MI (acute)	£4 344	Gamma	£96	Danese *et al*. 2016^45^
MI (year one+)	£944	Gamma	£135	Danese *et al*. 2016^45^
Stable AP (year one+)	£417	Gamma	£74	Danese *et al*. 2016^45^
Unstable AP (acute)	£2259	Gamma	£51	Danese *et al*. 2016^45^
Unstable AP (year one+)	£417	Gamma	£74	Danese *et al*. 2016^45^
HF (acute)	£280	Gamma	£105	Danese *et al*. 2016^45^
HF (year one+)	£1260	Gamma	£300	Danese *et al*. 2016^45^
ESRD (year one+)	£23 718	Gamma	£2372	Li *et al*. 2015^46^
RF RDN therapy	£6862	Gamma	£686	Microcosting 2022
Utilities				
Hypertension	1.00			
Stroke	0.63	Beta	0.04	Ward *et al*. 2007^47^
MI (months 1–6)	0.76	Beta	0.18	Ward *et al*. 2007^47^
MI (Months six+)	0.88	Beta	0.09	Henry *et al*. 2015^48^
Stable AP	0.81	Beta	0.02	Ward *et al*. 2007^47^
Unstable AP	0.77	Beta	0.04	Ward *et al*. 2007^47^
HF	0.68	Beta	0.01	Comin-Colet *et al*. 2012^49^
ESRD	0.72	Beta	0.37	Gorodetskaya *et al*. 2005^50^

Legend: SE, standard error; BP, blood pressure; RF RDN, radiofrequency renal denervation; MI, myocardial infarction; AP, angina pectoris; HF, heart failure; ESRD, end-stage renal disease; NICE, National Institute for Health and Care Excellence; NHS, National Health Service.

### Model validation

Model-projected event rates were compared with clinical outcomes reported in large-scale hypertension trials covering a broad range of patient populations/demographics and SBP reduction ranges.^37,51–59^ For these validation calculations, RRs of study-observed vs. analysis model-projected event rates were calculated. An RR >1.0 suggested under-projection of the analysis model, RR <1.0 overprediction, and an RR of 1.0 perfect concordance between the model and the study data. Further, lifetime model projections were compared with published lifetime incidences reported in epidemiological studies.^60–64^ Additionally, model projections were compared with those obtained from the QRISK3 calculator, which is used in the UK to assess risk of cardiovascular disease in the primary prevention setting.^65–67^

### Analysis outcomes and interpretation

The analysis outcomes were projected for 10-year and lifetime clinical events, RRs, costs, survival, quality-adjusted survival, and the resulting incremental cost-effectiveness ratio (ICER), which was calculated by dividing the incremental direct medical costs of treatment and sequelae by the incremental health benefits as expressed in quality-adjusted life years (QALYs). ICERs were reported as the mean values from probabilistic sensitivity analysis and were evaluated against the NICE cost-effectiveness thresholds of <£20 000 per QALY (cost-effective), £20 000–30 000 per QALY (potentially cost-effective), and >£30 000 per QALY (not cost-effective).

### Uncertainty and heterogeneity analysis

The effects of uncertainty and heterogeneity were examined through several analyses, and also reflected scenarios based on the large body of data available on RF RDN. First, one-way sensitivity analyses were completed by varying individual input parameters to 95% confidence intervals (*Table 1*) to determine which variables the ICER was most sensitive to. Second, multiway sensitivity and scenario analyses were completed by varying parameter sets to determine the demographics, baseline oSBP, and other assumptions whereby RF RDN was and was not cost-effective. Third, ICERs were calculated with both reductions against sham and reductions against baseline for the RF RDN cohort using the SPYRAL HTN-ON MED trial and HTN-OFF MED trial data. Fourth, sub-cohort analyses were completed for trial participants outside of the USA on three anti-hypertensives and for the aforementioned effect size estimates obtained from meta-analysis of sham-controlled randomised controlled trials (RCTs) of first- and second-generation RF RDN. Fifth, perspective was provided about the build-up of the lifetime ICER over time by calculating incremental costs, QALYs, and resulting ICERs also at 10, 15, and 20 years of follow-up. Finally, per guidance for health–economic analysis, a probabilistic sensitivity analysis was conducted for reductions against sham and baseline for the SPYRAL HTN-ON MED trial cohort, participants outside of the USA on three anti-hypertensives, assuming an effect size from meta-analysis of sham-controlled RCTs of first- and second-generation RF RDN, assuming oSBP reductions reported in HTN-OFF MED trial data, and assuming no distributions for demographic variables (which classify these variables as sources of model heterogeneity).^68^ These second-order Markov chain Monte Carlo simulations involved 10 000 repeated calculations for each analysis, randomly sampling from distributions of the input parameters in each analysis cycle (Supplementary material online, *S6*). Results were presented as a combined cost-effectiveness scatter plot and cost-effectiveness acceptability curve.

## Results

### Model validation

Model-projected stroke, MI, and death rates were in relative concordance with landmark hypertension clinical trial data and resulted in an average RR of 1.20, 1.31, and 0.98, respectively, indicating potential under-projection for stroke and MI of 20 and 31%, and over-projection of 2% for death (Supplementary material online, *S7*). The model was in relative concordance with lifetime epidemiological data for all modelled outcomes (Supplementary material online, *S8*), confirming that model-projected lifetime events are in keeping with reported lifetime event risks. Analysis model projections were reasonably comparable to those obtained from the UK QRISK3 calculator, again potentially under- rather than over-projecting relative to QRISK3 event projections (Supplementary material online, *S9*). On the basis of these validations, the risk functions in the analysis model were kept unadjusted for the analysis base case.

### Base case analysis

Over 10 years, clinical endpoint RRs for the RF RDN treated cohort vs. sham control were 0.80 for stroke, 0.88 for MI, 0.72 for HF, 0.89 for AP/CHD, 0.96 for ESRD, 0.84 for CVD, and 0.95 for ACD. These RRs were less pronounced over the lifetime horizon (*Table 2*). As RF RDN produced both costs and savings over the lifetime horizon, total lifetime costs for the base case were £24 486 for RF RDN and £19 723 for standard of care (+£4763). Total QALYs were 13.76 and 13.40 (+0.35 QALYs), yielding an ICER of £13 899 (deterministic: £13 482) per QALY. Cost savings with RF RDN resulted primarily from acute and follow-on costs for stroke, followed by HF and angina pectoris (Supplementary material online, *S10*).

**Table 2:** Base case results: clinical events over 10 years and lifetime, cost-effectiveness results over lifetime.

	10-year time horizon				Lifetime horizon			
Base case	SoC	RF RDN	Diff.	RR	SoC	RF RDN	Diff.	RR
Stroke	9.0%	7.2%	1.8%	0.80	33.8%	28.2%	5.7%	0.83
MI	7.5%	6.6%	0.9%	0.88	38.0%	37.4%	0.6%	0.99
AP/CHD	14.5%	12.9%	1.6%	0.89	28.0%	26.2%	1.9%	0.93
HF	5.0%	3.6%	1.4%	0.72	20.9%	16.3%	4.5%	0.78
ESRD	0.40%	0.40%	0.0%	0.96	1.03%	1.07%	0.04%	1.04
CVD	4.9%	4.2%	0.8%	0.84				
ACD	11.1%	10.5%	0.6%	0.95				
Costs					£19 723	£24 486	£4763	
QALYs					13.40	13.76	0.35	
ICER					£13 482 per QALY			

Legend: MI, myocardial infarction; AP, angina pectoris; CHD, coronary heart disease; HF, heart failure; ESRD, end-stage renal disease; CVD, cardiovascular death; ACD, all-cause death; QALYs, quality-adjusted life-years; ICER, incremental cost-effectiveness ratio; SoC, standard of care; RF RDN, radiofrequency renal denervation; Diff., difference; RR, relative risk.

### Uncertainty and heterogeneity analysis

**Figure 2:**
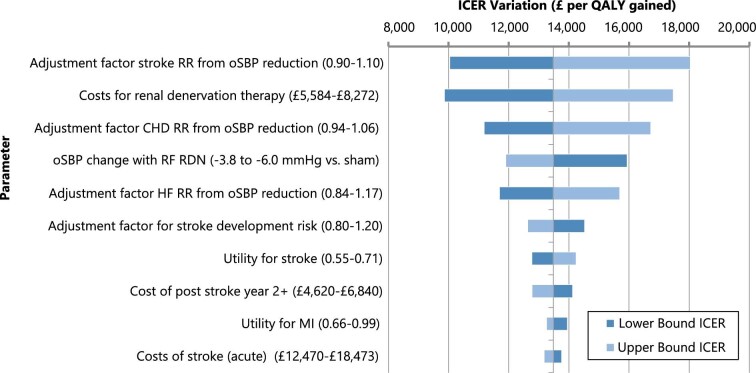
Tornado diagrame showing key parameters effecting ICER results. oSBP, office-based systolic blood pressure; CHD, coronary heart disease; MI, myocardial infarction; ICER, incremental cost-effectiveness ratio; RF RDN, radiofrequency renal denervation; RR, relative risk; QALY, quality-adjusted life year.

Analysis results were relatively insensitive across all scenario analyses. The reduction in costs for RF RDN and reduction in stroke and CHD from therapy effect had the greatest impact on the ICER (*Figure 2*). In sensitivity and scenario analysis, RF RDN remained cost-effective across a broad range of assumptions for cohort characteristics, cost/utility values, general population mortality rates, and therapy response rates (*Table 3*). For the SPYRAL HTN-ON MED trial cohort, the ICER for a reduction of 4.9 mmHg against sham was £13 899 per QALY. A reduction of 9.9 mmHg (the blood pressure reduction achieved against baseline) was £7979 per QALY. For −6.6 mmHg effect size observed vs. sham and −9.2 mmHg vs. baseline in HTN-OFF MED, the ICER was, respectively, £11 114 and £8456 per QALY gained. For the effect size observed for participants outside of the USA on three antihypertensive medications, the ICER was £10 321 per QALY. The pooled effect size of −4.8 mmHg vs. sham for first- and second-generation RF RDN devices, yielded an ICER of £14 165 per QALY, while the effect size of −5.7 mmHg based on second-generation devices only was £12 230 per QALY gained. See *Table 3* for further detail. Therapy benefits and the lifetime cost-effectiveness accrued over time, with theoretical shorter-term ICERs of £28 639, £19 790, and £15 812 per QALY gained at 10, 15, and 20 years. See Supplementary material online, *S12* for detail.

**Table 3:** Results of sensitivity and scenario analyses

		Costs (£)		QALYs				
	LYs gained (RF RDN vs. SoC)	RF RDN	SoC	RF RDN	SoC	ΔCosts	ΔQALYs	ICER (£ per QALY)
**SPYRAL HTN-ON MED trial**								
**Base Case** (oSBP effect size 4.9 mmHg vs. sham)	0.61	24 486	19 723	13.76	13.40	4763	0.35	13 482
oSBP effect size 9.9 mmHg (vs. baseline)	0.85	23 596	19 723	13.89	13.40	3873	0.49	7865
Subcohort on 3 antihypertensive medications treated outside the United States (oSBP effect size 6.9 mmHg vs. sham)	0.73	24 920	20 627	13.46	13.02	4293	0.44	9795
Treatment age 45 years	1.04	25 812	21 194	16.33	16.01	4618	0.32	14 300
Treatment age 70 years	0.53	20 469	15 210	8.73	8.44	5259	0.29	18 249
100% male	0.85	24 560	19 812	13.55	13.18	4748	0.36	13 050
100% female	0.78	24 509	19 690	14.82	14.53	4818	0.29	16 480
140 mmHg baseline oSBP	0.75	21 815	16 719	14.20	13.89	5096	0.31	16 406
180 mmHg baseline oSBP	0.88	25 757	21 120	13.50	13.13	4637	0.37	12 454
Costs (except RF RDN) 80% of base case assumption	0.61	20 961	15 778	13.76	13.40	5183	0.35	14 671
Costs (except RF RDN) 50% of base case assumption	0.61	15 674	9862	13.76	13.40	5813	0.35	16 454
Costs (except RF RDN) 120% of base case assumption	0.61	28 010	23 668	13.76	13.40	4343	0.35	12 293
RF RDN repeat procedure after 10 years	0.61	27 338	19 723	13.76	13.40	7615	0.35	21 555
Utilities 10% higher	0.61	24 486	19 723	14.05	13.72	4763	0.33	14 388
Utilities 10% lower	0.61	24 486	19 723	12.38	12.06	4763	0.32	14 980
Clinical event adjustment factor of 2.0	0.68	30 791	26 673	12.53	12.10	4117	0.42	9779
Clinical event adjustment factor of 0.6	0.48	20 620	15 359	14.50	14.23	5260	0.28	19 127
10% increased general population mortality	0.58	23 974	19 151	13.58	13.23	4823	0.34	14 087
10% decreased general population mortality	0.64	25 050	20 353	13.95	13.58	4697	0.36	12 869
Analysis horizon 15 years	0.27	21 672	16 755	12.89	12.64	4916	0.25	19 790
**OTHER STUDIES (Scenario analysis, see Supplementary material online, *S5* for meta-analysis details)**								
SPYRAL HTN-OFF MED (oSBP effect size 6.6 mmHg vs. sham)	0.73	24 479	20 055	14.84	14.46	4424	0.38	11 555
First- and second-generation RF RDN sham-controlled trials (pooled oSBP effect size 4.81 mmHg vs. sham)	0.60	24 502	19 723	13.75	13.40	4779	0.35	13 629
Second-generation RF RDN sham-controlled trials (pooled oSBP effect size 5.73 mmHg vs. sham)	0.65	24 334	19 723	13.78	13.40	4611	0.38	12 230

Legend: oSBP, office-based systolic blood pressure; SoC, standard of care; RF RDN, radiofrequency renal denervation; LY, life years (undiscounted); QALY, quality-adjusted life-year; ICER, incremental cost-effectiveness ratio.

**Figure 3:**
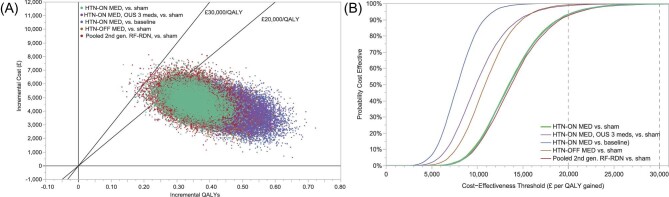
Probabilistic sensitivity analysis (PSA) scatterplot of results (*A*) and cost-effectiveness acceptability curve (CEAC) (*B*) for the base case (4.9 mmHg effect size, RF RDN vs. sham) and other key scenarios. OUS, outside United States; QALY, quality-adjusted life year; RF RDN, radiofrequency renal denervation.

The reduction against sham and baseline, respectively, yielded a 95% credibility interval of £7778–£22 831 per QALY and £4175 to £12 557 per QALY, with 93.4% and 100% of PSA simulations below the cost-effectiveness threshold of £20 000 per QALY. Across all scenarios, the probability that simulations were below the cost-effectiveness threshold of £20 000 per QALY ranged from 92.7% to 100%. See *Figure 3* and Supplementary material online, *S11*.

## Discussion

Multiple clinical consensus statements as well as the latest guidelines from the European Society for Hypertension now support the use of renal denervation as an additional therapeutic option in the treatment of hypertension.^3,11^ Decisions about therapy adoption and resource allocation require an in-depth understanding of how the blood pressure reductions gained with RF RDN might translate into long-term patient benefit and whether RF RDN treatment might be considered good value from a healthcare payer perspective.

The current study uses contemporary, established evidence associating blood pressure reduction with long-term reductions in clinical events to determine whether RF RDN provides sufficient benefit to justify its upfront cost of treatment. The analysis found that an effect size of 4.9 mmHg, as observed in the SPYRAL HTN-ON MED trial study, can be expected to lead to meaningful outcome improvement and related downstream cost savings in the long-term, which render the intervention good value for money. Specifically, a significant reduction of 20% for stroke and 12% for MI was found for a 10-year horizon in treated vs. control subjects, indicating the potential for RF RDN to reduce or delay major hypertension sequelae. RF RDN had a favourable cost-effectiveness profile across different studies, and a broad range of patient demographics and baseline systolic BPs, signalling potential therapeutic benefit and cost-effectiveness for a large proportion of the hypertensive population. While the contemporary study designs have evolved with the aim of overcoming limitations from the first-generation trials, including Symplicity HTN-2 and -3, sensitivity analyses using a pooled-effect estimate of sham-controlled studies of first- and second-generation RF RDN devices still yielded favourable cost-effectiveness results. The SPYRAL HTN-ON MED trial study, largely conducted during the COVID pandemic, observed unexpected levels of medication changes in the sham control group, potentially mitigating the observed RF RDN effect size against sham.^6^ Despite this, the trial demonstrates an effect size in oSBP that is cost effective, though the effect size assumed in the base case of the current study might be conservative given the difference between the effect size in the pilot and extension studies.

External validation demonstrated that modelled clinical events were approximately in line with, and likely conservative, compared with trial-observed data and lifetime risk epidemiological studies. Compared with the majority of trial and registry-observed event rates, the model seemed to under-project clinical events, indicating that the cost-effectiveness findings—again—might err on the conservative side. The analysis outcome was robust and not substantially altered across a broad range of uncertainty analyses, including Markov model structural changes and probabilistic sensitivity analyses, which yielded a very high likelihood that RF RDN was cost-effective.

A cost-effectiveness analysis published in 2014 based on the first randomized trial of RF RDN, HTN-2, found the therapy to be cost-effective at an ICER of £4805 per QALY gained. Importantly, that study and an earlier analysis for the USA assumed a much larger effect size of 32 mmHg from an open label study of resistant hypertension patients at baseline oSBP of 178 mmHg.^13,14^ It is likely that both studies underestimated the benefit of blood pressure reduction, as at the time—in the absence of the larger body of evidence now available from the more recent meta-regressions—the authors made a conscious decision to model clinical outcomes in both arms based on epidemiological functions rather than the application of RRs to the intervention arm. For example, a 32 mmHg reduction in oSBP projected a 10-year risk reduction of 0.70 for stroke, a value that is significantly more conservative than published meta-regression equations (including an RR of 0.47 for the Thomopoulos equations used in the current study).

Among the strengths of the current analysis are the extensive scenario and uncertainty analyses, which explicitly evaluate the effect of differing assumptions and—beyond the SPYRAL HTN-ON MED trial trial data used in the base case—also reflect the broader body of clinical evidence. At the same time, the study is subject to several limitations. First, while capturing a comprehensive set of health states, the model is still a simplification of reality. For example, the model does not capture repeat clinical events (such as myocardial infarctions or strokes) or a broader range of cardiovascular sequelae (such as arrhythmias, aortic aneurysms, or peripheral vascular disease). However, some of these effects are indirectly captured through post-event survival. Second, the model assumes that a constant effect size, based on 6-month results from the SPYRAL HTN-ON MED trial, is maintained over the subject's lifetime. This assumption seems supported by long-term treatment effects captured in 3-year data from earlier randomized studies,^69,70^ the GSR,^9,71^ and other recent data suggesting long-term durability of the RF RDN treatment effect out to beyond 8 years. Further, in most of these RF RDN trials, effect size tended to increase over time, suggesting the assumption of maintained 6-month effect size might be conservative. The same holds for potential added clinical benefit from reductions in diastolic, 24-h and night-time blood pressure or changes to dipping status that likely are not fully reflected when considering change in oSBP only. However, when modelling outcomes, office blood pressure is the preferred blood pressure measurement option, as these are the data we have available that detail long-term outcomes. Third, blood pressure was not modelled to increase with age in this lifetime analysis. However, as this potential effect would apply to both arms of the analysis model, the impact seems limited. At the same time, greater uptake of future drug strategies, such as polypills, or combination antihypertensive regimens—which again would benefit both strategies in the analysis—could balance potential blood pressure increase with age, supporting the chosen methodology. Fourth, this analysis only partially deals with stochastic (individual-level) uncertainty. While RF RDN is highly likely to be cost-effective at a population level (which is ultimately most relevant for healthcare payer decision-making), there is likely to be high individual variability in treatment effect and studies continue to examine which demographics, comorbidities, and ethnicities benefit most from RF RDN therapy. Fifth, while absolute event projections were based on widely established multivariate risk models, some uncertainty remains about their accuracy in event projection for the modelled cohort, and across a wide range of patient demographics and baseline blood pressures. In particular, the Framingham risk equations were derived from a sample of subjects with no prior cardiovascular disease, which differs from the modelled cohort of subjects from the SPYRAL HTN-ON MED trial. As the conducted validations with clinical trials have shown, the analysis model seems to under-project rather than over-project absolute clinical event rates, but not to a significant degree in either direction, supporting the choice of unadjusted risk equations in the base case. While variation in adjustment factors was shown to have a very limited effect on the ICER, the magnitude of absolute events avoided is directly impacted by the choice of adjustment factors. The calibration efforts may help to inform study-specific factors for future cohort-specific analyses, e.g. of the SYMPLICITY GSR study. Additionally, while out of scope of the current analysis, the potential effects of a broader future adoption of drugs such as SGLT2 inhibitors, which exhibit substantial cardiovascular event risk reduction, could modify population event rates and have implications for future clinical effectiveness analyses.^72,73^ Current RF RDN trials have relied on blood pressure change as the effectiveness measure. Reporting of clinical events and event reductions has previously only been modelled—based on event data observed in the single-arm GSR registry.^74^ Nevertheless, this is a usual limitation to cost-effectiveness analyses of hypertension interventions, which are commonly model-based.^43^ Sixth, the analysis relied on published regression equations to calculate clinical event reductions from changes in oSBP. The choice of the Thomopoulos equations for the current analysis was based on that study's designated focus of blood pressure lowering in hypertensive patients, which seems most appropriate for the current study. Other meta analyses included patients whereby the treatments given were for other conditions such as HF. Finally, the results are based on therapy effects reported from RF RDN treatment and therefore may not be generalizable to other RDN techniques.

## Conclusion

According to model-based projections of the SPYRAL HTN-ON MED trial and other contemporary evidence, catheter-based RF RDN can be expected to provide meaningful reductions in clinical event risks at an ICER substantially below the UK NICE cost-effectiveness thresholds, rendering RF RDN a cost-effective intervention for uncontrolled hypertension.

## Supplementary Material

qcae001_Supplemental_File

## Data availability

The underlying data informing the analysis are largely detailed in the main text and supporting supplementary material. However, further information can be made available upon reasonable request to the corresponding author.
