# Supplementary material for: Cost-effectiveness of catheter-based radiofrequency renal denervation for the treatment of uncontrolled hypertension: an analysis for the UK based on recent clinical evidence
Source: Eur Heart J Qual Care Clin Outcomes. 2024 Jan 9;10(8):698–708. doi: 10.1093/ehjqcco/qcae001 (PMC11656065; doi:10.1093/ehjqcco/qcae001)
Supplement: qcae001_Supplemental_File [file qcae001_supplemental_file.docx]

**SUPPLEMENTARY MATERIALS**

**Cost-Effectiveness of catheter-based radiofrequency renal denervation for the treatment of uncontrolled hypertension: An analysis for the UK based on recent clinical evidence**

Andrew S.P. Sharp, MBChB MD; Khoa N. Cao, MBBS MPH MS; Murray D. Esler, MBBS PhD; David E. Kandzari, MD; Melvin D. Lobo MBChB PhD; Roland E. Schmieder MD;

Jan B. Pietzsch, PhD

**S1: Structure of Markov Model**

Strategy-specific disease progression was modelled using a Markov state-transition model. This model consists of seven primary health states: hypertension alone, stroke, myocardial infarction (MI), angina pectoris (AP)/other coronary heart disease (CHD), heart failure (HF), end-stage renal disease (ESRD), and death.

All subjects commence in the hypertension state. As time progresses, subjects may develop a primary condition - stroke, MI, AP/ other CHD, HF, and ESRD or die. Subjects who enter MI or stroke will subsequently enter the post-MI or post-stroke state respectively to reflect the acute nature of the condition. After the development of a cardiovascular outcome, the development of further co-morbidities is possible as represented by the transitions to secondary health states. Subjects with MI may develop HF or stroke. Subjects with HF may develop stroke. Subjects with AP/other CHD may develop MI, stroke, or HF. Subjects with ESRD may develop AP/other CHD, MI, stroke, or HF.

**S2: Multivariate Risk Equations for Transitions between Health States**

The multivariate risk equations used to inform transition probabilities are represented in Table S.2.1 below. Mortality risks are provided in separate section S.3 below.

***Table S.2.1:*** Multivariate risk equations informing transitions between modelled health states

| **Transition** | **Equation** | **Source** |
| --- | --- | --- |
| Hypertension to CHD  (Female) | 1-EXP(-EXP((LN(YEARS)-(20.97 – 2.2449 * LN[Systolic BP] – 0.0621 * [Female Age] – 3.8522 * [Proportion in Menopause] + 0.0726 * [Female Age] * [Proportion in Menopause] – 0.6256 * LN[Triglycerides/HDL] – 0.0098 * [Proportion on Medications] * [200 – Systolic BP] * [(Systolic BP – 110)/100] – 0.5243 * [Proportion with Diabetes] – 0.3777 * [Proportion of Smokers] – 0.2688 * LN[Triglycerides] + 0.0529 * [Proportion of Alcohol Drinkers]))/[Scale Parameter for Weibull Survival Model for Women]))) | D'Agostino et al. 2000 (17) |
| Hypertension to CHD  (Male) | 1-EXP(-EXP((LN(YEARS)-(12.7868 – 1.0163 * LN[Systolic BP] – 0.0405 * [Male Age] – 0.9494 * LN[Triglycerides/HDL] – 0.161 * [Proportion on Medications] * [200 – Systolic BP] * [(Systolic BP – 110)/100] – 0.4412 * [Proportion with Diabetes] – 0.6042 * [Proportion of Smokers])/[Scale Parameter for Weibull Survival Model for Men]))) | D'Agostino et al. 2000 (17) |
| Hypertension to MI | 1-EXP(-((-LN(1-(1-EXP(LN(1-1/(1+EXP(-31.6879 + 0.0979 * [Age] – 0.0226 * [Triglycerides] + 5.7525 * LN[Triglycerides] – 0.4106 * [HDL] + 0.0151 * [LDL] + 0.014 * [Systolic BP] – 0.0257 * [GGT] + 0.7547 * [Proportion of Smokers] + 0.5827 * [Proportion with Diabetes] + 0.4154 * [Proportion with Cardiovascular Family History] + 0.0899 * [HDL] * LN[HDL] – 0.0004 * [Triglycerides] * [HDL] + 0.0001 * [Triglycerides] * [GGT])))))/10 | Voss et al. 2002 (20) |
| Hypertension to Stroke (Female) | 1-EXP(-((-LN(1-[Survival Value] ^ (EXP((0.0161 * [Systolic BP] + 0.00019 * [(Systolic BP – 110) * (200 – Systolic BP)] + 0.0488 * [Male Age] + 0.7864 * [Proportion with LVH] + 0.5224 * [Proportion of Smokers] + 0.5998 * [Proportion with AF] + 0.3429 * [Proportion with Diabetes]) – [M Value for Stroke for Women]))))) | Saposnik et al. 2008 (21), Andersen et al. 2005 (22), Wolf et al. 1991 (23) |
| Hypertension to Stroke (Male) | 1-EXP(-((-LN(1-[Survival Value] ^ (EXP((0.0152 * [Systolic BP] + 0.00026 * [(Systolic BP – 110) * (200 – Systolic BP)] + 0.0699 * [Female Age] + 0.8055 * [Proportion with LVH] + 0.5419 * [Proportion of Smokers] + 1.1173 * [Proportion with AF] + 0.5604 * [Proportion with Diabetes]) – [M Value for Stroke for Men]))))) | Saposnik et al. 2008 (21), Andersen et al. 2005 (22), Wolf et al. 1991 (23) |
| Hypertension to HF (Female) | 1-EXP(LN(1-(1/(1+EXP(ABS((-5.4997 + 0.0032 * [Systolic BP] + 0.0216 * [Female Age] + 1.0072 * [Proportion with LVH] - 0.0087 * [Female Vital Capacity] + 1.2454 * [Proportion with Valvular Disease] + 1.4275 * [Proportion with Diabetes] + 0.4792 * [Proportion with Cardiomegaly] – 0.9293 * [Proportion with Valvular Disease] * [Proportion with Diabetes] + 0.0092 * [Heart Rate]))))))/4 | Velgaleti et al. 2008 (18) |
| Hypertension to HF (Male) | 1-EXP(LN(1-(1/(1+EXP(ABS((-7.3611 + 0.0067 * [Systolic BP] + 0.0313 * [Female Age] + 0.8428* [Proportion with LVH] + 0.003 * [Female Vital Capacity] + 0.8868 * [Proportion with Valvular Disease] + 0.2383 * [Proportion with Diabetes] + 0.7968 * [Proportion with Cardiomegaly] + 0.0144 * [Heart Rate]))))))/4 | Velgaleti et al. 2008 (18) |

Table legend: CHD: Coronary Heart Disease, MI: Myocardial Infarction, HF: Heart Failure, HDL: High-Density Lipoprotein, BP: Blood Pressure, LDL: Low-Density Lipoprotein, GGT: Gamma-Glutamyl Transferase, LVH: Left Ventricular Hypertrophy, AF: Atrial Fibrillation

**S3: Mortality Transitions**

Health-state specific probabilities of death were modelled as follows: For patients in the hypertension state, age- and gender-specific mortality from the latest UK lifetables was assumed. All other model states featured event-specific mortality that was further separated into acute and long-term mortality where appropriate. Secondary health states utilized the higher mortality rate of the two conditions to avoid double-counting. See Table S3.1 for details.

***Table S3.1:*** Condition-specific mortality.

| **Condition** | **Methodology** | **Value or Range** | **Source** |
| --- | --- | --- | --- |
| **Stroke (30 Days)** | Uniform mortality rate | 0.101 | Wafa et al. 2020 (25) |
| **Stroke (Annual)** | Uniform hazard ratio applied to general population mortality | 2.3 | Geisler et al. 2012 (14) |
| **MI (30 Days)** | Age-stratified mortality rate | 0.2386-0.3703 | Asaria et al. 2017 (26) |
| **MI (Annual)** | Age and gender-stratified hazard ratio applied to general population mortality | 1.313-3.338 | Smolina et al. 2012 (27) |
| **AP/Other CHD (Annual)** | Average across stable AP, unstable AP, and other CHD, with an age and gender-specific scaling modifier | 0.0125-0.2187 | Rapsomaniki et al. 2014 (28), Buckley et al. 2009 (29) |
| **Heart Failure (Annual)** | Age-stratified mortality rate | 0.0353-0.0956 | Taylor et al. 2019 (30) |
| **ESRD (Annual)** | Age-stratified mortality rate | 0.015-0.097 | 24^th^ UK Renal Registry Annual Report, 2022 (31) |

Table legend: MI: Myocardial Infarction, AP: Angina Pectoris, CHD: Coronary Heart Disease, ESRD: End-Stage Renal Disease.

**S4: Meta-Regression Equations for Clinical Event Relative Risk Reductions**

The meta-regression equations for clinical event risk reductions from Thomopoulos et al. 2014 (32) are represented in the table below (expressed as relative risks (RR)). Standard errors are also represented, which were utilized in the probabilistic sensitivity analysis.

***Table S4.1:*** Meta-regression equations associating reduction in office systolic blood pressure with event risk reductions (derived from Thomopoulos et al., 2014 (32))

| **Condition** | **Equation** | **Standard Error** |
| --- | --- | --- |
| CHD | RR=e^(-0.092436 - 0.0098787 * [oSBP Reduction])^ | 0.033 |
| Stroke | RR=e^(-0.120685 - 0.0200016 * [oSBP Reduction])^ | 0.050 |
| HF | RR=e^(-0.130645 - 0.0299026 * [oSBP Reduction])^ | 0.085 |

Table legend: CHD: Coronary Heart Disease, HF: Heart Failure, oSBP: Office Systolic Blood Pressure, RR= Relative Risk.

**S5: Methods to Derive Pooled Renal Denervation Effect Size for Scenario Analysis**

The pooled effect size was obtained from a meta-analyses of RF RDN sham-controlled randomised controlled trials in patients with uncontrolled hypertension, with or without concomitant anti-hypertensive medication use. The basis for the meta-analyses was a systematic review, with a protocol published on the PROSPERO register of systematic reviews (34-36).

Trial data from intention-to-treat analyses (where available, modified intention-to-treat or per-protocol if not available) and last follow-up time (before cross-over) were used for the analyses. All analyses were conducted in R (version 4.2.1). Results of the analyses are presented below for all trials (Analysis One) and only 2nd generation trials (Analysis Two).

***Figure S5.1:*** Analysis One: Forest plot of pair-wise meta-analysis results of sham-controlled randomised controlled trials of radiofrequency renal denervation (1^st^ and 2^nd^ generation).


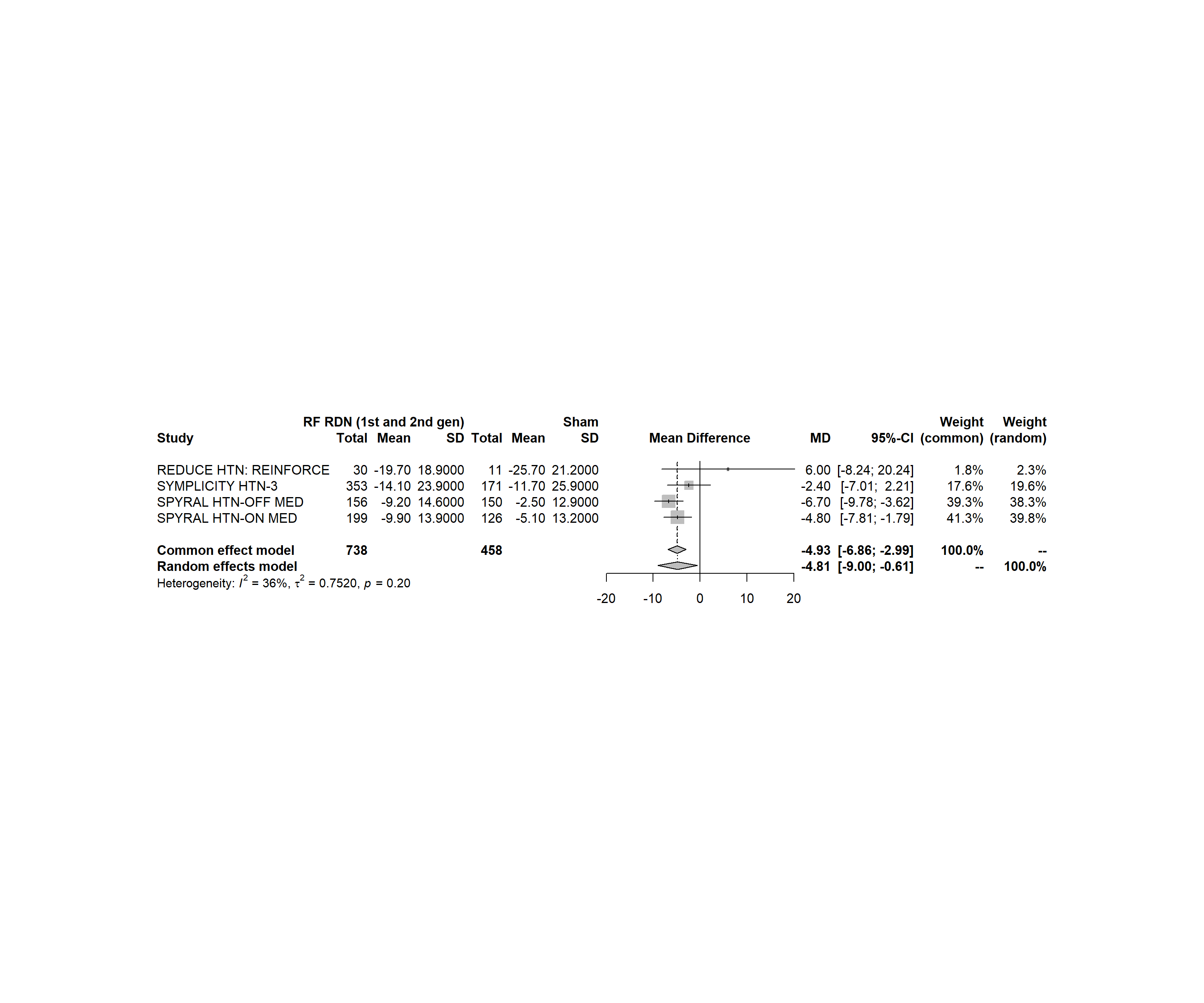


The low level of heterogeneity (Cochrane’s Q p-value = 0.20; I^2^ = 36%) indicated that the common effects model is, statistically, an appropriate model to use. However, due to known clinical heterogeneity, caused by different patient populations and follow-up times, the random effects model was deemed more appropriate to use in this context. The random effects model generated a pooled mean difference of -4.81 (95% CI: -9.00 to -0.61) for the mean change in office SBP from baseline between radiofrequency renal denervation (1^st^ and 2^nd^ generation) and sham. There were no outliers identified. All studies in this indication reported 95% confidence intervals which lay within the 95% confidence interval of the pooled effect.

***Figure S5.2:*** Analysis Two: Forest plot of pair-wise meta-analysis results of sham-controlled randomised controlled trials of radiofrequency renal denervation (2^nd^ generation only).


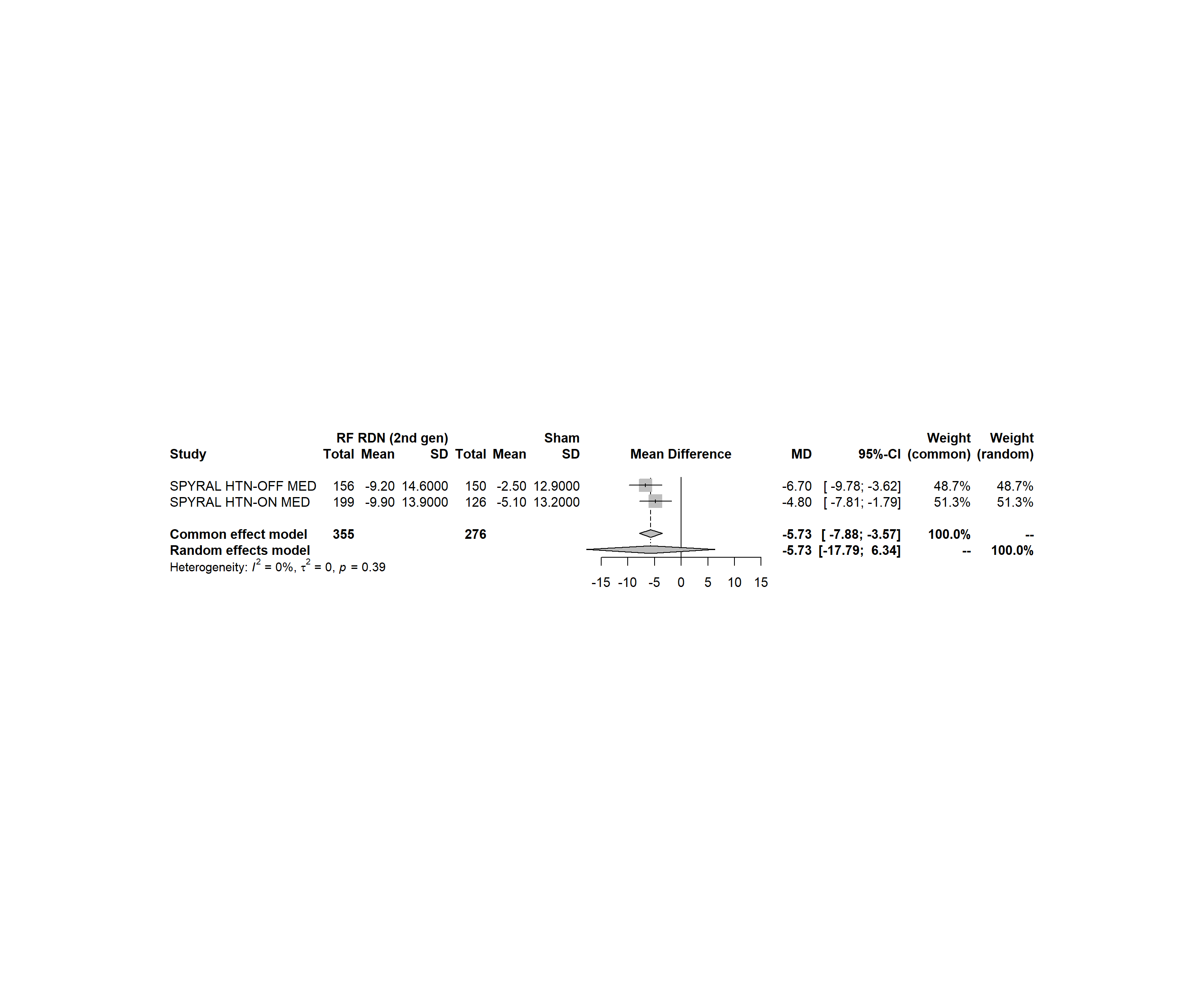


In this analysis, the low level of heterogeneity (Cochrane’s Q p value = 0.39; I^2^ = 0%) indicated that the common effects model was, statistically, an appropriate model to use. Due to the limited number of studies available, the confidence interval for the random effects model appeared implausible given both studies are significantly different individually. Therefore, the common effects model was deemed to be more appropriate in this instance. The common effects model generated a pooled mean difference estimate of -5.73 (95% CI: -7.88 to -3.57) for the mean change in SBP from baseline between RF RDN (2^nd^ generation) and sham. No outliers were identified. All studies in this indication reported 95% confidence intervals which lay within the 95% confidence interval of the pooled effect.

**S6: Details on Probability Distributions used in Probabilistic Sensitivity Analysis**

Table S6.1 below shows details on the probability assumptions used for probabilistic sensitivity analysis. While variation of cohort characteristics informed by underlying trial data may or may not be included in probabilistic sensitivity analysis (with guidelines for the UK advising to keep these inputs deterministic as cohort characteristics are ‘known’ to the analyst), the authors opted to include distributional information to reflect uncertainty in event risk transitions which is informed by Framingham and other risk score functions for which no distributional information is available. In any case, maintaining the clinical values as deterministic inputs – which was explored in a scenario analysis - led to essentially unchanged PSA findings, with only a slightly narrower distribution of the ICER values computed from simulation runs.

***Table S6.1:*** *Distributional information of key variables modelled in probabilistic sensitivity analysis.*

| **Parameter** | **Value** | **SD** | **SE** | **Lower Bound** | **Upper Bound** | **Distribution** |
| --- | --- | --- | --- | --- | --- | --- |
| Age | 55.0 years | 9.7 | 0.53 | 54.0 | 56.0 | Normal |
| Gender (% Female) | 19.9% |  | 0.022 | 15.8% | 24.3% | Beta |
| Baseline Systolic BP | 163 mmHg | 7.3 | 0.40 | 162.2 | 163.8 | Normal |
| Treatment Effect 1 | 4.9 mmHg | 10 | 0.54 | 3.8 | 6.0 | Normal |
| Treatment Effect 2 | 9.9 mmHg | 10 | 0.54 | 8.8 | 11.0 | Normal |
| **Costs (Monthly)** | | | |  |  |  |
| Hypertension (Year one+) | £23 | - | 2.3 | £19 | £28 | Gamma |
| RF RDN Therapy | £6,862 | - | 686 | £5,584 | £8,272 | Gamma |
| Stroke (Acute) | £15,327 | - | 1,533 | £12,470 | £18,473 | Gamma |
| Stroke (Year one) | £902 | - | 90 | £734 | £1,088 | Gamma |
| Stroke (Year two+) | £473 | - | 47 | £385 | £570 | Gamma |
| MI (Acute) | £4,344 | - | 96 | £4,158 | £4,533 | Gamma |
| MI (Year one+) | £86 | - | 11 | £65 | £109 | Gamma |
| Stable AP (Year one+) | £35 | - | 6 | £24 | £48 | Gamma |
| Unstable AP (Acute) | £2,259 | - | 51 | £2,161 | £2,360 | Gamma |
| Unstable AP (Year one+) | £35 | - | 6 | £24 | £48 | Gamma |
| HF (Acute) | £280 | - | 105 | £113 | £521 | Gamma |
| HF (Year one+) | £105 | - | 25 | £62 | £159 | Gamma |
| ESRD (Year one+) | £1,977 | - | 198 | £1,608 | £2,382 | Gamma |
| **Utilities** | | | |  |  |  |
| Stroke | 0.63 | - | 0.04 | 0.55 | 0.71 | Beta |
| MI (Months one to six) | 0.76 | - | 0.18 | 0.72 | 0.79 | Beta |
| MI (Months six+) | 0.88 | - | 0.09 | 0.66 | 0.99 | Beta |
| Stable AP | 0.81 | - | 0.02 | 0.77 | 0.85 | Beta |
| Unstable AP | 0.77 | - | 0.04 | 0.69 | 0.84 | Beta |
| HF | 0.68 | - | 0.01 | 0.65 | 0.71 | Beta |
| ESRD | 0.72 | - | 0.37 | 0.00 | 1.00 | Beta |
| **Adjustment Factors (for risk equations)** | | | | | | |
| Stroke risk equation | 1.00 | - | - | 0.80 | 1.20 | Uniform |
| MI risk equation* | 0.90 | - | - | 0.72 | 1.08 | Uniform |
| CHD risk equation | 1.00 | - | - | 0.80 | 1.20 | Uniform |
| HF risk equation | 1.00 | - | - | 0.80 | 1.20 | Uniform |
| ESRD risk equation | 1.00 | - | - | 0.80 | 1.20 | Uniform |
| Stroke risk ratio from treatment effect | 1.00 | - | - | 0.90 | 1.10 | Normal |
| CHD risk ratio from treatment effect | 1.00 | - | - | 0.94 | 1.06 | Normal |
| HF risk ratio from treatment effect | 1.00 | - | - | 0.83 | 1.17 | Normal |

Table legend: BP: Blood Pressure, RF RDN: Radiofrequency Renal Denervation, MI: Myocardial Infarction, AP: Angina Pectoris, CHD: Coronary Heart Disease, HF: Heart Failure, ESRD: End-Stage Renal Disease, SE: Standard Error, SD: Standard Deviation

*: For MI risk equation, the male-only PROCAM equation was adjusted based on data from the Tromsø Study to project MI risk for the mixed-gender HTN-ON MED cohort. Therefore, the base case value is 0.90 as opposed to 1.0.

**S7: Model Validation One – Comparison of Model Projections to Study-observed Data**

To assess whether the model’s clinical endpoint projections were reasonably in line with existing clinical trial and registry data, large-scale clinical trials and registries within the hypertension field were identified, the model was populated with the respective study’s reported cohort characteristics, and it was assessed how the resulting model projections compared to the stroke, MI and all-cause death rates reported in the respective studies. Where lipid levels were not reported, a standard set of lipid levels derived from a hypertension cohort in NHANES was utilised (24) The model utilised UK lifetable data for mortality projections (42).

In brief, the clinical trials and registries span a range of notable large-scale drug and intensive BP control studies from 2000-2021, covering a total of 69,210 patients. Mahfoud et al. reported the outcomes of 2,652 patients from the Global Symplicity Registry for renal denervation (37). The VALUE trial analysed the outcomes of 7,080 patients treated with either valsartan or amlodipine (51, 52). The ACCOMPLISH trial followed 11,506 patients treated with benazepril plus amlodipine or hydrochlorothiazide (53). ON-TARGET examined 8,576 patients randomised to telmisartan, ramipril or a combination of both (54). TRANSCEND followed 5,926 patients assigned to telmisartan or placebo (55). HOPE examined the effect of ramipril in 9,500 patients (56). From an intensive BP control perspective, ACCORD examined the effects of intense BP control in 4,733 diabetic patients, STEP examined in 9,624 hypertensive patients and SPRINT in 9,631 high-risk patients (57-59).

For each study and the evaluated endpoints, the relative risk (study-observed vs. model-projected) was calculated, and an average of this relative risk across all of the investigated studies calculated. Hence, a relative risk greater than 1.0, corresponded to a situation where model-projected were lower than study-observed event rates (purple values in the table), while a relative risk of less than 1.0 corresponded to an instance where the model-projected events were higher than the study-reported events, i.e., the model would overpredict the event. By extension, risk functions in the model could be multiplied (or, ‘adjusted’) by the respective relative risk to achieve resemblance of model-projected events and study-reported events.

The results are shown in Table S7.1. For stroke, RRs were larger than 1.0 in seven cohorts (hence, the model under-projected events), and less than 1.0 for four cohorts. The average RR was 1.20. For myocardial infarction, the model under-projected for eight cohorts and over-projected for three cohorts, with RRs ranging from 0.70 to 3.08 (average RR=1.31). For all-cause death, the model under-projected for six cohorts and over-projected for five cohorts, with RRs ranging from 0.23 to 1.46 (average RR=0.68). Notably, the STEP study, which examined intensive vs. standard therapy in Chinese patients with hypertension, reported a significantly lower mortality rate compared to other studies. Nonetheless, the model under-projected for all three conditions (stroke, MI, all-cause death) for six trial cohorts, and over-projected for all three conditions for only one trial cohort (SPRINT).

***Table S7.1A-D:*** Comparisons of model projected event rates with observed clinical study and registry data. (A) Cohort Characteristics, (B) Stroke Projections, (C) MI Projections, and (D) All-Cause Death.

A.

| Cohort characteristics | **Age** | **Gender (%male)** | **Diabetes (%)** | **Current smoker** | **Hypertension** | **CHD/CVD** | **Baseline oSBP** | **oSBP (on treatment)** | **Study follow-up (yrs.)** | **Reference** |
| --- | --- | --- | --- | --- | --- | --- | --- | --- | --- | --- |
| GSR R-HTN | 61 | 57.5% | 43.0% | 32.0% | 100.0% | 48.0% | 175 | 153.5 | 3 |  |
| GSR T2D | 64 | 59.0% | 100.0% | 32.0% | 100.0% | 28.0% | 165 | 150 | 3 | Mahfoud, 2020 (37) |
| VALUE Valsartan cohort | 67 | 58.0% | 33.0% | 22.0% | 100.0% | 44.0% | 155 | 142 | 4.2 | Julius, 2004 (51) |
| VALUE Monotherapy | 67 | 59.0% | 30.0% | 26.0% | 100.0% | 48.0% | 151 | 137 | 3 | Julius, 2006 (52) |
| SPRINT Standard trtmt. | 68 | 65.0% | 0.0% | 13.0% | 100.0% | 20.0% | 140 | 136 | 3.2 | SPRINT, 2015 (58) |
| ACCOMPLISH | 61 | 61.0% | 60.2% | 11.4% | 100.0% | 48.8% | 145.4 | 132.5 | 3 | Jamerson, 2008 (53) |
| ACCORD | 62 | 52.3% | 100.0% | 13.2% | 100.0% | 33.7% | 139.4 | 134 | 4.7 | Cushman, 2010 (57) |
| ONTARGET Ramipril subgroup | 66 | 72.8% | 36.7% | 12.4% | 100.0% | 74.4% | 141.8 | 136 | 5 | ONTARGET, 2008 (54) |
| TRANSCEND | 67 | 57.4% | 35.6% | 9.7% | 100.0% | 74.3% | 141.3 | 141 | 4.7 | TRANSCEND, 2008 (55) |
| HOPE  Placebo arm | 66 | 74.2% | 38.0% | 14.5% | 46.1% | 81.4% | 139 | 139 | 4.5 | HOPE, 2000 (56) |
| STEP Standard trtmt. | 66 | 46.1% | 19.4% |  | 100.0% | 6.4% | 146 | 137 | 3.3 | Zhang, 2021 (59) |
| AVERAGE | 65.0 | 60.2% | 45.1% | 18.6% | 95.1% | 46.1% | 149.0 | 140.0 |  |  |

Table legend: oSBP: Office-Based Systolic Blood Pressure, HR: Hazard Ratio, CHD: Coronary Heart Disease, CVD: Cardiovascular Disease, yrs: Years.

B.

| **STROKE** | Study-observed | Model-projected | RR (study vs. model) |
| --- | --- | --- | --- |
| GSR R-HTN | 4.8% | 2.3% | *2.09* |
| GSR T2D | 4.0% | 3.6% | *1.11* |
| VALUE Valsartan cohort | 4.2% | 3.7% | *1.12* |
| VALUE Monotherapy | 2.3% | 2.3% | *0.99* |
| SPRINT Standard trtmt. | 1.4% | 2.0% | *0.71* |
| ACCOMPLISH | 2.3% | 1.5% | *1.53* |
| ACCORD | 2.7% | 3.6% | *0.74* |
| ONTARGET Ramipril subgroup | 4.7% | 3.6% | *1.32* |
| TRANSCEND | 4.6% | 4.1% | *1.13* |
| HOPE  Placebo arm | 6.1% | 3.6% | *1.69* |
| STEP Standard trtmt. | 1.7% | 2.2% | *0.78* |
| AVERAGE |  |  | *1.20* |

Table Legend: trmt: treatment, RR: Relative Risk.

C.

| **MI** | Study-observed | Model-projected | RR (study vs. model) |
| --- | --- | --- | --- |
| GSR R-HTN | 2.3% | 1.9% | *1.21* |
| GSR T2D | 4.0% | 3.3% | *1.21* |
| VALUE Valsartan cohort | 4.8% | 4.0% | *1.19* |
| VALUE Monotherapy | 2.7% | 2.6% | *1.04* |
| SPRINT Standard trtmt. | 2.3% | 2.9% | *0.81* |
| ACCOMPLISH | 2.8% | 1.5% | *1.87* |
| ACCORD | 6.4% | 5.2% | *1.23* |
| ONTARGET Ramipril subgroup | 4.8% | 3.9% | *1.23* |
| TRANSCEND | 5.0% | 6.2% | *0.81* |
| HOPE  Placebo arm | 12.3% | 4.0% | *3.08* |
| STEP Standard trtmt. | 1.9% | 2.7% | *0.70* |
| AVERAGE |  |  | *1.31* |

Table Legend: trmt: treatment, MI: Myocardial Infarction, RR: Relative Risk.

D.

| **All-cause death** | Study-observed | Model-projected | RR (study vs. model) | General population per lifetables (England) | HR Study vs. general population |
| --- | --- | --- | --- | --- | --- |
| GSR R-HTN | 5.7% | 3.9% | *1.46* | 2.5% | 2.28 |
| GSR T2D | 7.1% | 6.3% | *1.13* | 3.1% | 2.29 |
| VALUE Valsartan cohort | 11.0% | 10.0% | *1.10* | 5.9% | 1.86 |
| VALUE Monotherapy | 6.2% | 6.4% | *0.97* | 3.1% | 1.99 |
| SPRINT Standard trtmt. | 4.2% | 6.6% | *0.64* | 4.5% | 0.93 |
| ACCOMPLISH | 4.5% | 3.8% | *1.18* | 2.4% | 1.88 |
| ACCORD | 6.0% | 9.3% | *0.64* | 4.6% | 1.29 |
| ONTARGET Ramipril subgroup | 11.8% | 9.5% | *1.25* | 7.0% | 1.69 |
| TRANSCEND | 11.7% | 12.6% | *0.93* | 6.7% | 1.75 |
| HOPE  Placebo arm | 12.9% | 10.0% | *1.29* | 6.3% | 2.05 |
| STEP Standard trtmt. | 1.5% | 6.4% | *0.23* | 3.9% | 0.38 |
| AVERAGE |  |  | *0.98* |  | 1.67 |

Table Legend: trmt: treatment; HR: Hazard ratio, RR: Relative Risk.

Overall, these validation results indicate that the model is in reasonably close keeping with study-observed data, and that maintaining the source risk functions (including Framingham equations) without applying an adjustment factor seemed appropriate for the base case analysis.

**S8: Model Validation Two - Comparison of model projections to published lifetime event incidence**

To assess whether lifetime events projected through the model were in close concurrence with published lifetime incidence reported in epidemiological studies, several large studies across stroke, MI, CVD, HF, and ESRD were identified. The analysis model was populated with each cohort’s reported characteristics and subsequently ran over the remaining lifetime of the cohort. The cumulative model-projected incidence was then compared to the study-reported incidence.

1. **Stroke**

According to values reported by the Global Burden of Disease Study in 2016, the model is in keeping for a cohort of patients with a blood pressure (BP) of 145mmHg (29.6% in model vs. 26% for age 55, 26.0% vs 24% for age 65, 21.8% vs 22% for age 75) (60). For an age group of 65 and a BP of 164mmHg, the model is also in relative keeping with estimates from the Framingham study as reported by Seshadri et al (15.2% in model vs. 11.5% at ten years, and 30.5% vs. 23% at lifetime) (61).

1. **Myocardial Infarction**

Based on 33-year values reported on the Tromsø study, the model’s annual incidence of myocardial infarction for an assumed office systolic BP (oSBP) of 150mmHg is in close keeping with real-world values (1.1% in model vs 1.2% for males aged 65, 0.6% vs 0.7% for females aged 65, 2.1% vs 2.3% in males aged 75, 1.2% vs 1.3% in females aged 75, 2.8% vs 4.4% in males aged 85, 1.6% vs 2.4% in females aged 85) (62).

1. **Heart Failure**

In a study of nearly 40,000 participants followed for 18 years on average, Huffman et al. reported a lifetime risk of heart failure of 21% for 45-year-old males (baseline oSBP of 143mmHg) and 16% for 45-year-old females (baseline oSBP of 138 mmHg) (63). This is in keeping with the model’s predicted lifetime risk of 20.0% and 12.4% for 45-year-old males and females respectively with the same baseline oSBP as reported in the study.

1. **End-Stage Renal Disease**

Turin et al. reported the lifetime risk of ESRD over a follow-up of 25,985,361 person-years (64). In 40-year-old males with an eGFR ≥ 60 (which corresponds to the HTN-ON MED cohort), the predicted lifetime risk of ESRD was 0.99%, which is in keeping with the lifetime risk of 1.20% for a cohort with the same demographics.

Together with the short-term comparisons against clinical study-reported outcomes, the findings of the lifetime validations suggested reasonable concurrence between projected values and expected real-world values.

**S9: Model Validation 3 - Comparison of Model Projections to QRISK3 Risk Predictions**

QRISK3 is a set of updated algorithms based on a sample of UK primary care data to quantify the absolute risks of CVD in a population aged 25-84, which include established and new risk factors (65). UK NICE has previously recommended QRISK3 to assess cardiovascular risk in the primary prevention setting (66).

To compare projections of the current model to those of QRISK3, several comparison calculations were conducted using the QRISK®3-2018 risk calculator (67). For these calculations, the following parameters were set to zero in QRISK3 as these parameters were neither variables in the analysis model nor reported in the SPYRAL studies - severe mental illness, CKD stage, migraines, rheumatoid arthritis, systemic lupus, atypical antipsychotic meds, steroid tablets, and erectile dysfunction.

Analyses were run for a cohort resembling the HTN-ON MED pilot cohort (age 53 years, 16% female, on hypertensive medications) and included the following: (1) A cohort with diabetes, atrial fibrillation, a family history of MI and currently smoking (moderate smoker for QRISK3); (2) A cohort with atrial fibrillation, family history of MI and currently smoking (moderate smoker for QRISK3); (3) A cohort with a family history of MI and currently smoking (moderate smoker for QRISK3); (4) A cohort with a family history of MI; (5) A cohort with no risk factors other than elevated oSBP.

Relative risks (QRISK3 projection vs. analysis model) were calculated for the combination of MI and stroke endpoints. RRs ranged from 0.83 for the lowest risk cohort (group 5) to 1.57 for the highest risk cohort (group 1). These findings suggest there is reasonable agreement between QRISK3-projected events and the set of equations utilized in the current study (Framingham and PROCAM) and further suggest the current model might under- rather than over-project stroke and MI.

***Table S9.1:*** Comparisons of model-projected combined stroke and MI event rates to projections obtained from UK QRISK3 risk calculator (analyses for several defined cohorts ranging from high risk factors to low risk factors).

| COHORT: Age 53, 16% female, SBP 164 mmHg, on hypertensive meds | | | | | | | | |
| --- | --- | --- | --- | --- | --- | --- | --- | --- |
| Diabetes, Afib, Family history of MI, Current smoker (moderate for Qrisk) | | | | | | | | |
| **RF RDN Model Ten-Year Risk** | MI | 14.20% | Stroke | 28.3% | Sum | 42.50% |  |  |
| **Qrisk3: Ten-year risk MI or stroke** | Male | 66.10% | Female | 69.50% | Combined | 66.64% | RR (Qrisk3 vs. analysis model) | **1.57** |
| Afib, Family history of MI, Current smoker (moderate for Qrisk) | | | | | | | | |
| **RF RDN Model Ten-Year Risk** | MI | 9.10% | Stroke | 22.00% | Sum | 31.10% |  |  |
| **Qrisk3: Ten-year risk MI or stroke** | Male | 42.40% | Female | 38.80% | Combined | 41.82% | RR (Qrisk3 vs. analysis model) | **1.34** |
| Family history of MI, Current smoker (moderate for Qrisk) | | | | | | | | |
| **RF RDN Model Ten-Year Risk** | MI | 9.50% | Stroke | 12.60% | Sum | 22.10% |  |  |
| **Qrisk3: Ten-year risk MI or stroke** | Male | 24.00% | Female | 14.20% | Combined | 22.43% | RR (Qrisk3 vs. analysis model) | **1.02** |
| Family history of MI | | | | | | | | |
| **RF RDN Model Ten-Year Risk** | MI | 4.90% | Stroke | 8.10% | Sum | 13.00% |  |  |
| **Qrisk3: Ten-year risk MI or stroke** | Male | 14.80% | Female | 7.90% | Combined | 13.70% | RR (Qrisk3 vs. analysis model) | **1.05** |
| No risk factors other than elevated SBP of 164 mmHg | | | | | | | | |
| **RF RDN Model Ten-Year Risk** | MI | 3.30% | Stroke | 8.10% | Sum | 11.40% |  |  |
| **Qrisk3: Ten-year risk MI or stroke** | Male | 10.20% | Female | 5.50% | Combined | 9.45% | RR (Qrisk3 vs. analysis model) | **0.83** |

Table Legend: Afib: atrial fibrillation. MI: myocardial infarction, RF RDN: radiofrequency renal denervation, SBP: systolic blood pressure, RR: relative risk.

**S10: Cost Breakdown by Treatment Strategy**

Figure S10.1 below provides a breakdown of the strategy-specific costs by health state. A reduction in clinical event costs were observed for the renal denervation group compared to the standard of care cohort. Cost savings with RF RDN resulted primarily from acute and follow-on costs for stroke, followed by HF and AP.

***Figure S10.1:*** Costs by cost type, by strategy (lifetime).

**S11: Results of Probability Sensitivity Analysis**

Table S11.1 provides additional PSA results to complement those shown in the body of the manuscript. Pooled estimate refers to the effect size calculated from the meta-analysis of second-generation RF RDN trials (-5.7 mmHg oSBP change vs. sham control). The base case assuming no distributions for cohort characteristics is shown at the bottom. It closely resembles the base case PSA for which cohort characteristics were included as PSA input distributions in an effort to evaluate the effect of uncertainty in the multivariate risk equations (Framingham, PROCAM).

***Table S11.1:*** Results of probabilistic sensitivity analyses.

| **Scenario** | **Credibility Interval Lower Limit** | **Credibility Interval Upper Limit** | **Cost-Effectiveness Probability** |
| --- | --- | --- | --- |
| **Against Sham** | £7,778 | £22,831 | 93.4% |
| **Against Baseline** | £4,175 | £12,557 | 100.0% |
| **Pooled Estimate** | £8,043 | £23,056 | 92.7% |
| **Outside US with 3 Antihypertensives** | £5,158 | £18,035 | 98.8% |
| **HTN-OFF MED** | £6,342 | £18,171 | 99.0% |
| **Base case, assuming no distributions for cohort characteristics** | £7,943 | £22,655 | 93.5% |

**S12: Time Horizon-specific Analysis**

While guidelines for cost-effectiveness analysis, including the UK NICE guide to the methods of technology appraisal, specify the lifetime analysis horizon as appropriate, understanding how this lifetime cost-effectiveness builds up over time can be useful to decision makers. Below, we provide ICER projections for 10, 15, and 20 years, in addition to the lifetime results.

***Table S12.1:*** Build-up of the lifetime cost-effectiveness over time: Incremental costs, QALYs, and resulting ICERs at 10, 15, and 20 years, and over lifetime.

|  | Cost difference | QALY difference | Resulting ICER |
| --- | --- | --- | --- |
| 10 years | £5,243 | 0.18 | £28,639/QALY |
| 15 years | £4,916 | 0.25 | £19,790/QALY |
| 20 years | £4,766 | 0.30 | £15,812/QALY |
| Lifetime | £4,763 | 0.35 | £13,482/QALY |

**S13: Consolidated Health Economic Evaluation Reporting Standards (CHEERS) checklist**

Table S12.1 below provides a completed CHEERS checklist based on CHEERS 2022 reporting guidance (16).

***Table S13.1:*** Completed CHEERS checklist.

| **Section / Topic** | **Item No.** | **Guidance for Reporting** | **Reported in Section** |
| --- | --- | --- | --- |
| **Title** |  |  |  |
| Title | 1 | Identify the study as an economic evaluation and specify the interventions being compared. | Title, page 1, lines 1-2. Identifies the RF RDN cost-effectiveness analysis. |
| Abstract |  |  |  |
| Abstract | 2 | Provide a structured summary that highlights context, key methods, results, and alternative analyses. | Abstract. Pg. 2, lines 1-191. The abstract is structured and includes aims, methods and results, and a conclusion. |
| **Introduction** |  |  |  |
| Background and objectives | 3 | Give the context for the study, the study question, and its practical relevance for decision making in policy or practice. | Introduction, pg. 4, lines 1-25 and pg. 5, lines 1-15. |
| **Methods** |  |  |  |
| Health economic analysis plan | 4 | Indicate whether a health economic analysis plan was developed and where available. | Methods, pg. 5, lines 18-24 and pg. 6, lines 1-9. The decision-analytic Markov model and health states modelled are introduced. |
| Study Population | 5 | Describe characteristics of the study population (such as age range, demographics, socioeconomic, or clinical characteristics). | Methods, Clinical Data subsection, pg. 7, lines 20-22; also summarized in Table 1, pg. 28-29. |
| Setting and Location | 6 | Provide relevant contextual information that may influence findings. | NA |
| Comparators | 7 | Describe the interventions or strategies being compared and why chosen. | Methods, Clinical Data subsection, pg. 7, lines 11-25 and pg. 8, lines 1-8; Treatment effect assumptions are also summarized in Table 1, pg. 28-29. |
| Perspective | 8 | State the perspective(s) adopted by the study and why chosen. | Methods, Model Structure and Framework subsection. Pg. 6, lines 11-12 detail the analysis was conducted from the perspective of a UK NHS payer. |
| Time horizon | 9 | State the time horizon for the study and why appropriate. | Methods, Model Structure and Framework subsection, pg. 6, line 12. |
| Discount Rate | 10 | Report the discount rate(s) and reason chosen. | Methods, Model Structure and Framework subsection, pg. 6, line 13. |
| Selection of Outcomes | 11 | Describe what outcomes were used as the measure(s) of benefit(s) and harm(s). | Methods, Analysis Outcomes and Interpretation subsection, pg. 9, lines 5-11 |
| Measurement of outcomes | 12 | Describe how outcomes used to capture benefit(s) and harm(s) were measured | Methods, Analysis Outcomes and Interpretation subsection, pg. 9, lines 5-11 |
| Valuation of outcomes | 13 | Describe the population and methods used to measure and value outcomes. | Methods, Transition Probabilities and Relative Risk Reductions subsection, pg. 6, lines 22-24 and pg. 7, lines 1-9.  Methods, Analysis Outcomes and Interpretation subsection, pg. 9 lines 8-11 |
| Measurement and valuation of resources and costs | 14 | Describe how costs were valued. | Methods, Costs and Health-Related Quality of Life subsection, pg. 8, lines 10-13 |
| Currency, price date, and conversion | 15 | Report the dates of the estimated resource quantities and unit costs, plus the currency and year of conversion. | Methods, Costs and Health-Related Quality of Life subsection, pg. 8, lines 13-15. |
| Rationale and description of model | 16 | If modelling is used, describe in detail and why used. Report if the model is publicly available and where it can be accessed. | Methods, pg. 5, lines 18-24 and pg. 6 lines 1-9-. The decision-analytic Markov model and health states modelled are introduced. Also: Methods, Model Structure and Framework subsection, pg. 6, lines 11-20. |
| Analytics and assumptions | 17 | Describe any methods for analysing or statistically transforming data, any extrapolation methods, and approaches for validating any model used. | Detailed in part in previously documented sections.  Methods. Model Validation subsection, pg. 8, lines 20-24 and pg. 9, lines 1-3. |
| Characterizing heterogeneity | 18 | Describe any methods used for estimating how the results of the study vary for subgroups | Methods, Uncertainty and Heterogeneity Analysis subsection, pg. 9, lines 13-24 and pg. 10, lines 1-8. |
| Characterizing distributional effects | 19 | Describe how impacts are distributed across different individuals or adjustments made to reflect priority populations. | Methods, Uncertainty and Heterogeneity Analysis subsection, pg. 9, lines 13-24 and pg. 10, lines 1-8. |
| Characterizing uncertainty | 20 | Describe methods to characterize any sources of uncertainty in the analysis. | Methods, Uncertainty and Heterogeneity Analysis subsection, pg. 9, lines 13-24 and pg. 10, lines 1-8. |
| Approach to engagement with patients and others affected by the study | 21 | Describe any approaches to engage patients or service recipients, the general public, communities, or stakeholders (such as clinicians or payers) in the design of the study. | NA |
| Results |  |  |  |
| Study parameters | 22 | Report all analytic inputs (such as values, ranges, references) including uncertainty or distributional assumptions. | Table 1. pg. 28-29. |
| Summary of main results | 23 | Report the mean values for the main categories of costs and outcomes of interest and summarize them in the most appropriate overall measure | Results, Base Case Analysis subsection, pg. 10, lines 21-24 and pg. 11, lines 1-4.  Table 2, pg. 30. |
| Effect of uncertainty | 24 | Describe how uncertainty about analytic judgments, inputs, or projections affects findings. Report the effect of choice of discount rate and time horizon, if applicable. | Results, Model Validation subsection, pg. 10, lines 11-19.  Results, Uncertainty and Heterogeneity Analysis subsection, pg. 11, lines 6-25.  Table 3, pg. 31-32. |
| Effect of engagement with patients and others affected by the study | 25 | Report on any difference patient/service recipient, general public, community, or stakeholder involvement made to the approach or findings of the study | NA |
| Discussion |  |  |  |
| Study findings, limitations, generalizability, and current knowledge | 26 | Report key findings, limitations, ethical or equity considerations not captured, and how these could affect patients, policy, or practice. | Discussion, pg. 12-15, lines 1-25, lines 1-25, lines 1-26, and lines 1-16, respectively. The discussion section clearly details the key results, limitations, and implications of this analysis. |
| Other Relevant Information |  |  |  |
| Source of funding | 27 | Describe how the study was funded and any role of the funder in the identification, design, conduct, and reporting of the analysis | Funding. Pg. 16, line 2. |
| Conflicts of interest | 28 | Report authors conflicts of interest according to journal or International Committee of Medical Journal Editors requirements. | Conflicts of Interest. Pg. 1, lines 4-13. |
